# Design and analysis of three-dimensional printing of a porous titanium scaffold

**DOI:** 10.1186/s12891-021-04520-1

**Published:** 2021-08-02

**Authors:** Jiajie Yang, Yaqiang Li, Xiaojian Shi, Meihua Shen, Kaibing Shi, Lingjie Shen, Chunxi Yang

**Affiliations:** 1 Nantong Haimen People’s Hospital, 1201 Beijing Road, Haimen District, Nantong City, 226100 Jiangsu Province China; 2 Renji Hospital Affiliated to Shanghai Jiaotong University School of Medicine, 145 Shandong Zhong Lu, Shanghai, 200001 China

**Keywords:** Bone defect, Metal scaffold, Three-dimensional printing, Titanium, Safety factor, Compressive strength

## Abstract

**Objective:**

Mechanic strength, pore morphology and size are key factors for the three-dimensional (3D) printing of porous titanium scaffolds, therefore, developing optimal structure for the 3D printed titanium scaffold to fill bone defects in knee joints is instructive and important.

**Methods:**

Structural models of titanium scaffolds with fifteen different pore unit were designed with 3D printing computer software; five different scaffold shapes were designed: imitation diamond-60°, imitation diamond-90°, imitation diamond-120°, regular tetrahedron and regular hexahedron. Each structural shape was evaluated with three pore sizes (400, 600 and 800 μm), and fifteen types of cylindrical models (size: 20 mm; height: 20 mm). Autodesk Inventor software was used to determine the strength and safety of the models by simulating simple strength acting on the knee joints. We analyzed the data and found suitable models for the design of 3D printing of porous titanium scaffolds.

**Results:**

Fifteen different types of pore unit structural models were evaluated under positive pressure and lateral pressure; the compressive strength reduced when the pore size increased. Under torsional pressure, the strengths of the imitation diamond structure were similar when the pore size increased, and the strengths of the regular tetrahedron and regular hexahedron structures reduced when the pore size increased. In each case, the compressive strength of the regular hexahedron structure was highest, that of the regular tetrahedron was second highest, and that of the imitation diamond structure was relatively low. Fifteen types of cylindrical models under a set force were evaluated, and the sequence of comprehensive compressive strength, from strong to weak was: regular hexahedron > regular tetrahedron > imitation diamond-120° > imitation diamond-90° > imitation diamond-60°. The compressive strength of cylinder models was higher when the pore size was smaller.

**Conclusion:**

The pore size and pore morphology were important factors influencing the compressive strength. The strength of each structure reduced when the pore size (400, 600 and 800 μm) increased. The models of regular hexahedron, regular tetrahedron and imitation diamond-120°appeared to meet the conditions of large pore sizes and high compressive strength.

## Introduction

Bone defects are common in the clinic and are usually associated with diseases, such as infection, osteolysis, original implant loosening or tumor excision. Clinically, bone loss has been addressed with methods such as cement, autogenous bone grafts, and artificial implants [1, 2]. However, autogenous bone grafts are painful and source-limited, and are accompanied with complications, such as donor site morbidity [3]. Bone cement can lead to complications of absorption poisoning, bone absorption poisoning, bone absorption and allergies [4]. In recent years, artificial implant materials, such as calcium phosphate [5], ceramic [6], polymer materials [7] and metal [8], have been developed. Among them, metal is used in the clinic because of its high strength, high load capacity, shape memory, inertness and superelasticity. Common metal scaffolds include tantalum, titanium and titanium-nickel alloy.

Tantalum is a biocompatible material with good tissue compatibility with human tissue, causing almost no side effects. Therefore, this metal is widely used in medical clinics [9, 10]. However, the tantalum scaffold is expensive, and its strong oxidation and high melting point lead to costly and difficult processing. The production cost is high, and the methods are limited, and the tantalum powder shape is irregular; therefore, it is difficult to use 3D printing to generate tantalum scaffolds. Titanium has very high corrosion resistance, low density, and the highest strength / weight ratio of metals in addition to being non-magnetic. Titanium and titanium alloy materials also have good biomechanical properties and biocompatibility. Therefore, titanium is widely used in orthopedic implants [11].

3D printing technology, which is based on computer-aided design data, uses powder chromatography as a layer-by-layer printing method to quickly create objects with a complex 3D structure [12]. Compared with the traditional production technology, such as porous scaffolds, 3D printing technology has greater advantages in the control of the scaffold porosity, pore size, pore volume, spatial arrangement and other surface properties [13]. 3D printing technology has been used in many industries, including the medical industry, such as for orthopedic surgery and bone defects treatment, to make models and help doctors better understand complex anatomy and pathology [14–18]. Porous titanium scaffolds generated with 3D printing have great advantages for the control of porosity, pore size, pore volume, spatial arrangement and other surface features. 3D printing of porous titanium metaphyseal cones has previously been applied to revision TKA [19] and 3D printing of porous titanium is conducive to bone differentiation and new bone formation [20–22].

At present, there are some problems in the 3D porous titanium scaffolds. For example, its porous structure will reduce its mechanical properties. Different pore shapes will affect its mechanical properties. And different pore sizes will also affect the mechanical properties and the growth of bone tissue. Reasonable design of porous structure can not only promote the regeneration and growth of bone tissue, but also acquire well mechanical properties. Therefore, the pore shape and pore size are very important for the design of 3D porous titanium scaffolds. Several studies have done this work. One study has designed 3D porous titanium scaffolds with five different pore shapes and the same pore size, and found that under the condition of near porosity and the same pole sectional size, the 3D structure with different unit cells has a great impact on scaffold strength [23]. Another study has designed six types of 3D porous titanium scaffolds, and found the compressive stiffness values covered the range of cortical and trabecular bone [24]. However, these studies did not comprehensively compare the mechanical properties of scaffolds with different pore sizes and the same pore shapes or with different pore shapes and the same pore sizes under the positive, lateral and torsional pressure. This study designed five different pore shapes, and three different pore sizes for each pore shape, and finite element analysis was carried out under the positive, lateral and torsional pressure. The results were converted into force or a safety factor. The design models with large pore sizes and high compressive strength were screened out, which could provide references for the further production of porous titanium scaffolds with the 3D printing technique.

## Materials and methods

### Scaffold model design

Ti6Al4V has good heat resistance, strength, plasticity, toughness, formability, corrosion resistance and biocompatibility [25, 26]. Ti6Al4V was used as the material used for titanium scaffolds, and its main physical properties include: mass density 4.43 g/cc, yield tensile strength 880 MPa, ultimate tensile strength 950 MPa, modulus of elasticity 113.8 GPa, compressive yield strength 970 MPa, Poisson’s ratio 0.342 ul, shear strength 44 GPa [27–29]. Ti6Al4V powder has an average size of 45 μm [30].

Some studies have designed 3D porous titanium scaffolds with different pore shapes of imitation diamond, regular tetrahedron, regular octahedron, three circles type [23] and regular hexahedron [21, 25, 31]. Many studies suggested that large pore size was beneficial to the growth of bone tissue, such as 500 [24] and 700 μm [32]. Therefore, this study chose five common unit structures: imitation diamond-60°, imitation diamond-90°, imitation diamond-120°, regular tetrahedron and regular hexahedron and three pore sizes: 400, 600 and 800 μm.

**Fig. 1:**
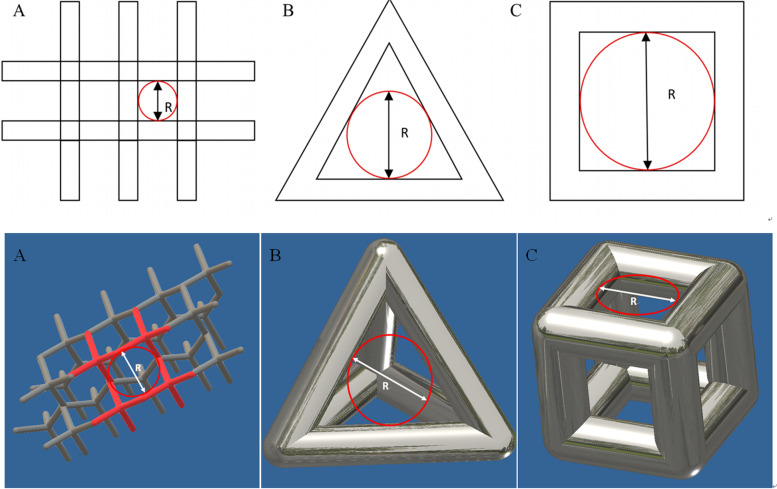
**A** Overall top view of the imitation diamond unit structure; R is the size of the aperture. **B** Regular tetrahedron unit structure has a diameter R in either side. **C** Size of the aperture R is the inscribed circle diameter on either side of the regular hexahedral unit structure

Five types of unit structures were designed using Autodesk Inventor software (Inventor 2016, Autodesk Inc. San Rafael, California, USA), imitation diamond-60°, imitation diamond-90°, imitation diamond-120°, regular tetrahedron and regular hexahedron. The size of the bracket bar was 400 μm. Each type of unit structure has three pore sizes: 400, 600 and 800 μm, and the aperture was set as shown in Fig. 1. The fifteen kinds of unit structures were aligned to obtain a cylinder with a size of 20 mm and a height of 20 mm (cylinder size and height were finely adjusted for overall integrity).

### Finite element analysis

The positive pressure, lateral pressure and torsional pressure were applied to fifteen kinds of unit structures and the corresponding fifteen kinds of cylindrical models with ABAQUS software (ABAQUS 2016, Simulia Inc. Providence, Rhole Island, USA). Finite element analysis was carried out to obtain the mechanical properties and compare the data. These simulations and collation took 3 months approximately.

The configuration of the computer used to run the Autodesk Inventor software and the ABAQUS software included a CPU model: Intel Core i7 6700HQ, quad-core eight threads, 256 GB solid state + 1 TB hard disk, 16G memory, NVIDIA GeForce GTX 1060 graphics card, and Win10 system.

#### Setting and analysis of unit structure model

**Fig. 2:**
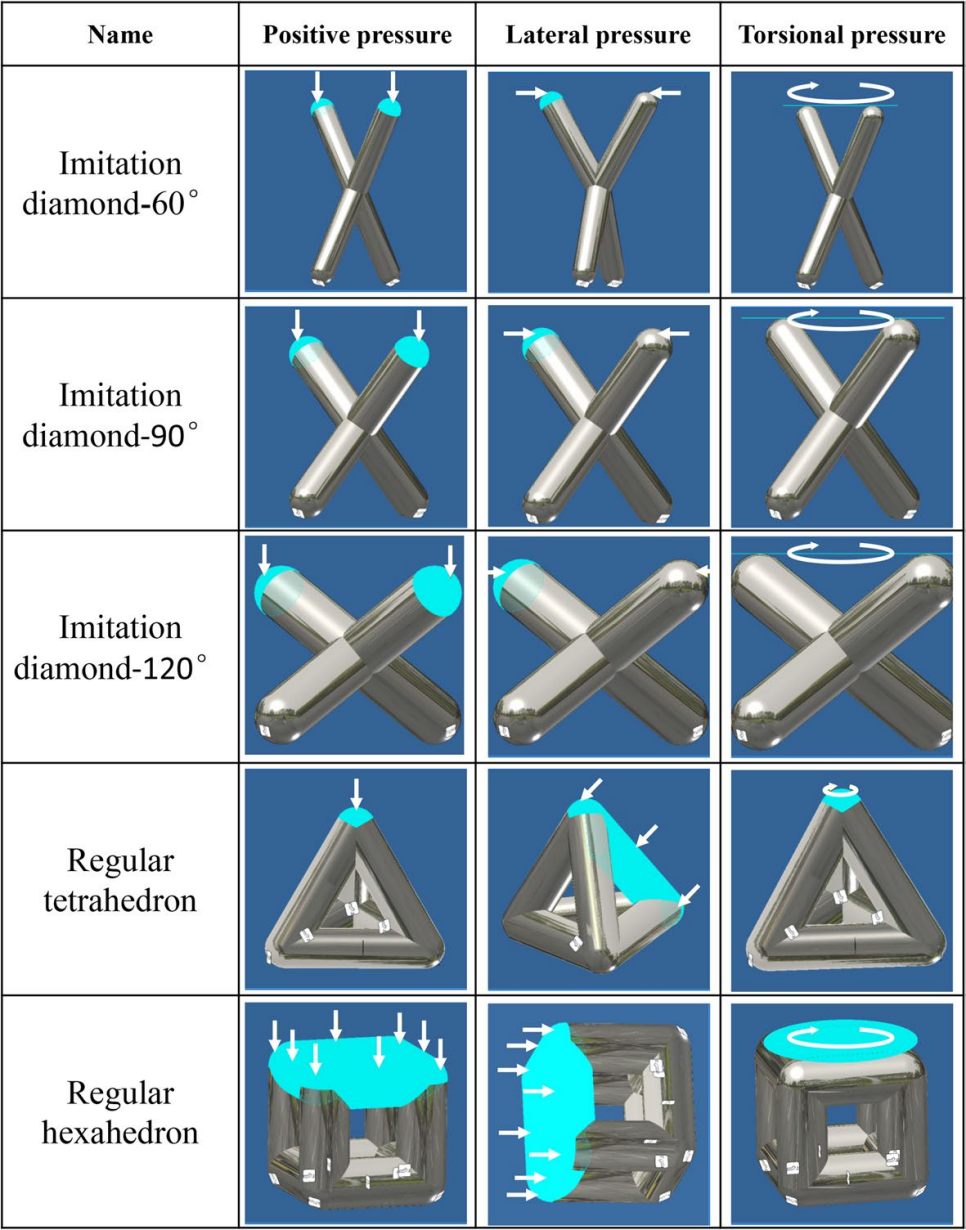
Schematic of different unit structures and applied pressure

When the positive pressure, lateral pressure and torsional pressure were applied to the fifteen kinds of unit structures, and when the minimum safety factor of the model was greater than or equal to 1, the force was the maximum pressure the unit structure could bear (accurate to 0.5 N). When the unit structure was under positive pressure, lateral pressure and torsional pressure, a fixed surface was selected and a force was applied to the offside of the fixed side. The unit structure and force are shown in Fig. 2. The maximum forces of each unit structure in the safe state obtained by software analysis were compared.

#### Safety factor

**Fig. 3:**
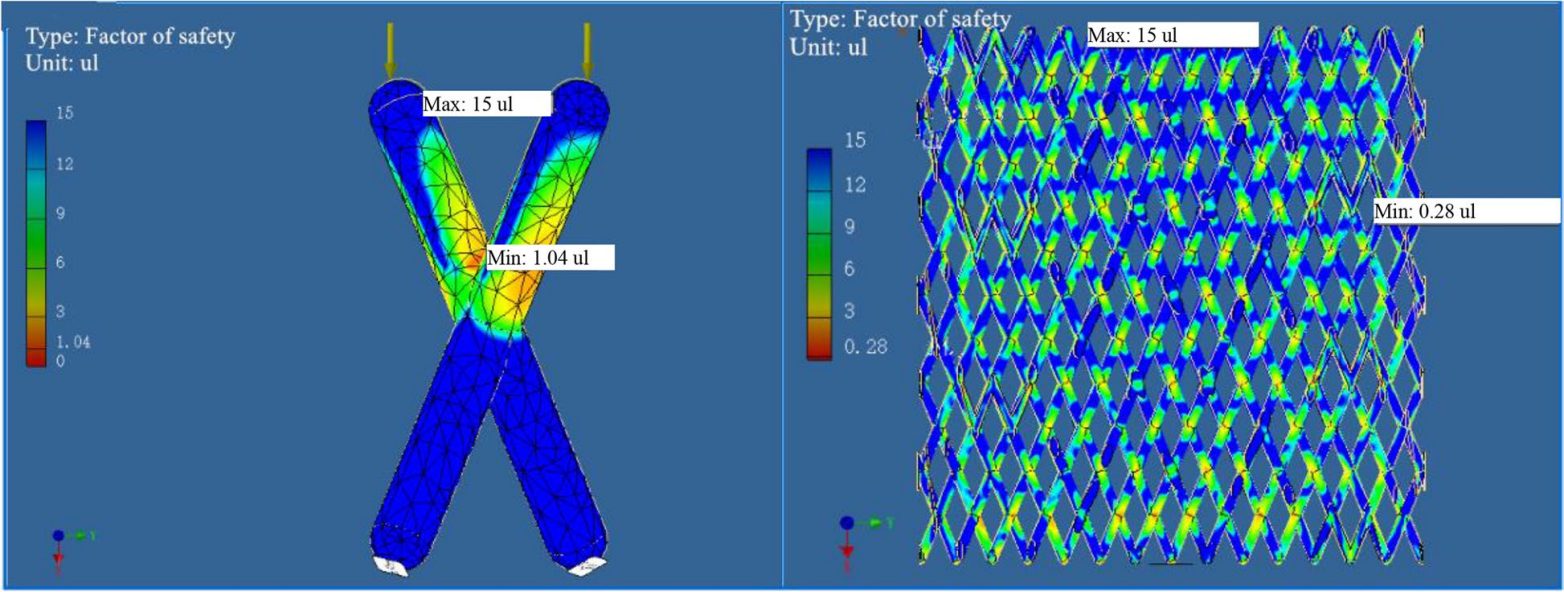
Unit structure and cylindrical structure of imitation diamond-60°under lateral pressure (600 N); blue indicates security, and red indicates danger. The reddest point occurred when the safety factor was the smallest

The safety factor is the ratio of the ultimate stress to the allowable stress. The safety factor is the strength margin, considering factors that accurately calculate the load and stress, the importance of the work of the machine, and the reliability of the material. The value is greater than or equal to 1 (less than 1 indicates permanent deformation) [33, 34]. When the computer simulated the applied force, the point with the minimum value of the safety factor of models (Fig. 3) was determined, which was the point where the model was most likely to be damaged.

#### Setting and analysis of cylindrical model

**Fig. 4:**
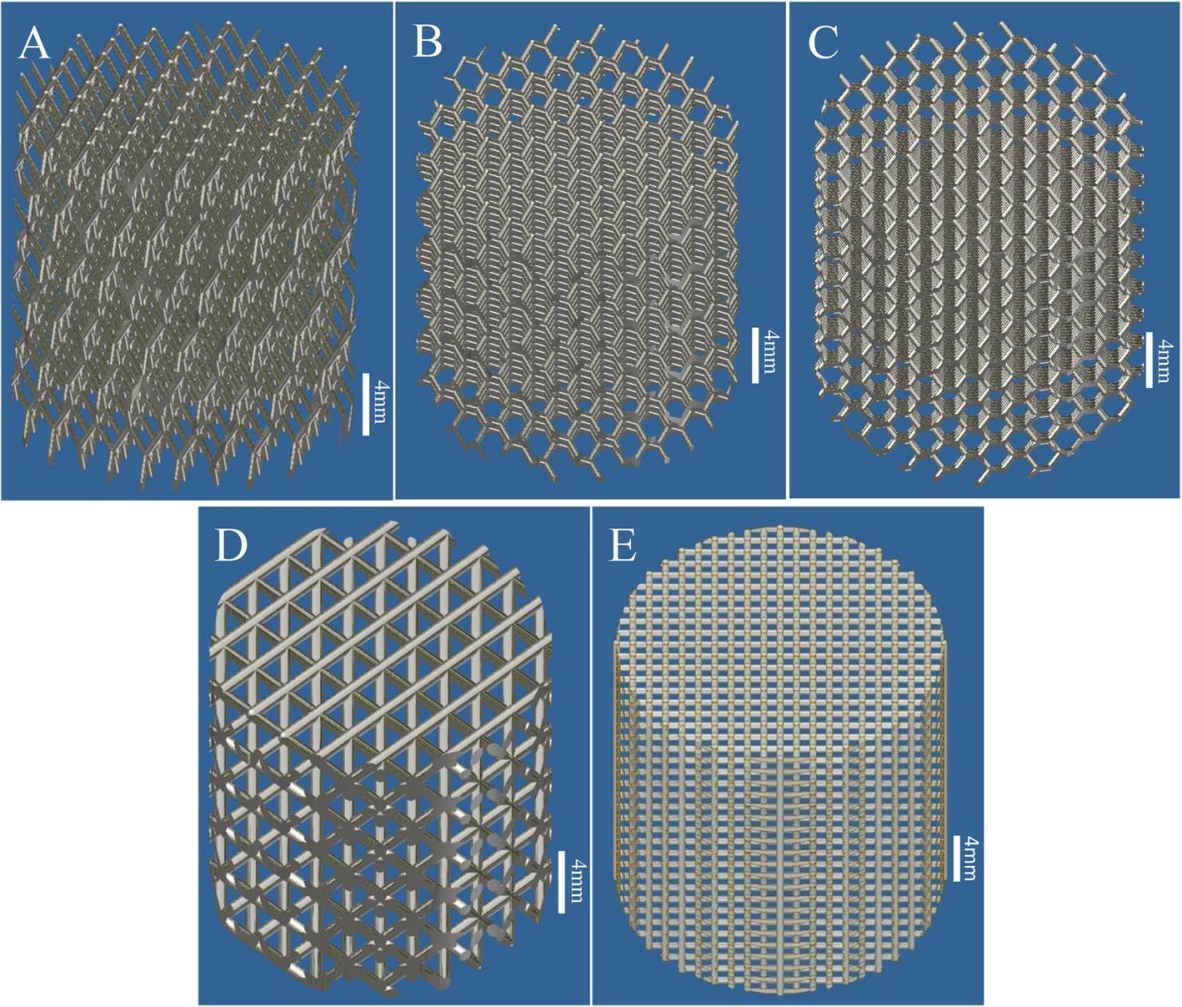
The five types of cylindrical model. **A** Imitation diamond-60°; **B** imitation diamond-90°; **C** imitation diamond-120°; **D** regular tetrahedron; **E** hexahedron

Five types of cylindrical models are shown in Fig. 4. The pressure applied to the fifteen kinds of cylindrical models was set such that the pressure on the knee was approximately twice the weight of the adult (approximately 60 kg). When an adult is standing on one leg, the knee is subjected to a stress of approximately 3 ~ 4 times the person’s weight. The pressure on the knees when climbing stairs is about 3 to 6 times of body weight [35]. When the person kicks the ball, the twisting force is approximately 3000–4000 N. Therefore, when the positive, lateral and torsional pressures were applied to the fifteen kinds of cylindrical models, they were applied with 1 times, 3 times and 5 times the positive pressure and 1 times and 3times the lateral pressure, and the torsional pressure applied was 2000, 3000 and 4000 N. Then, the safety factors were obtained using the ABAQUS software analysis under different values for various forces and compared.

## Results and analysis

### Results and analysis of unit structure model

**Fig. 5:**
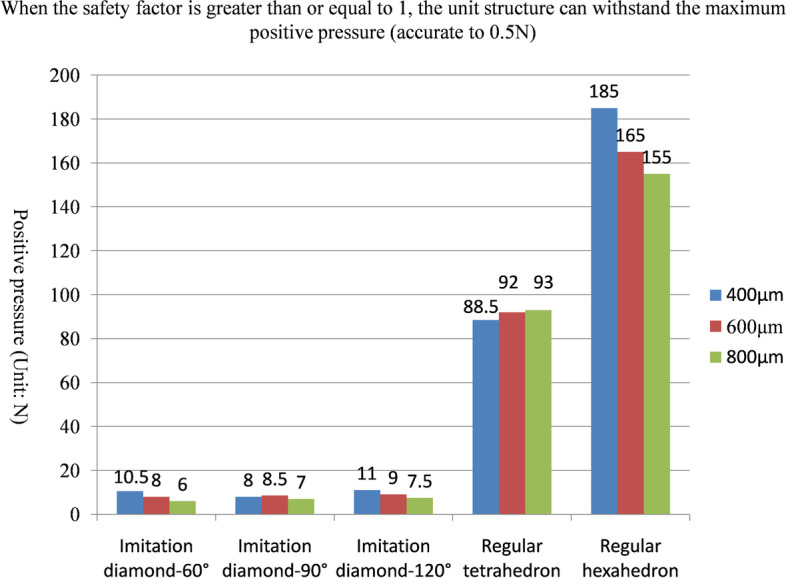
When the safety factor is greater than or equal to 1, the unit structure can withstand the maximum positive pressure (accurate to 0.5 N)

**Fig. 6:**
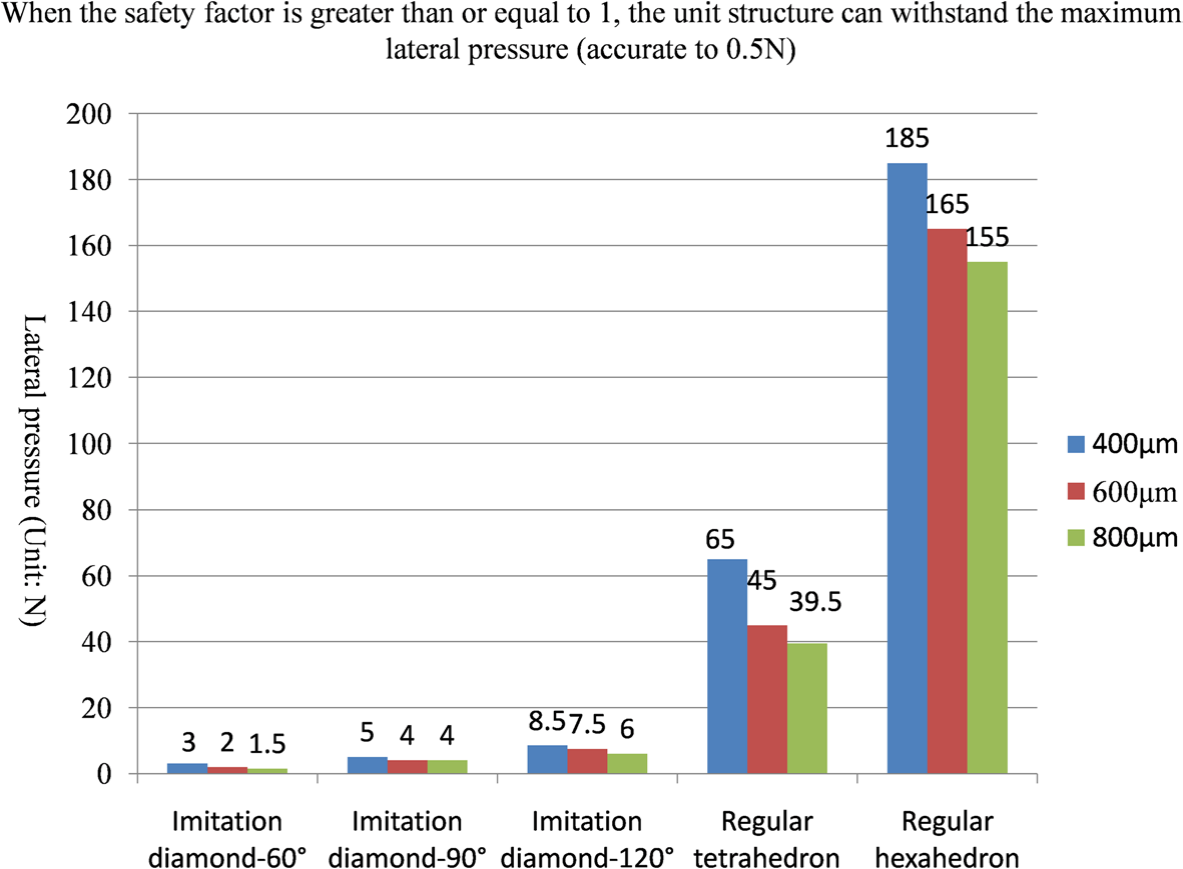
When the safety factor is greater than or equal to 1, the unit structure can withstand the maximum lateral pressure (accurate to 0.5 N)

**Fig. 7:**
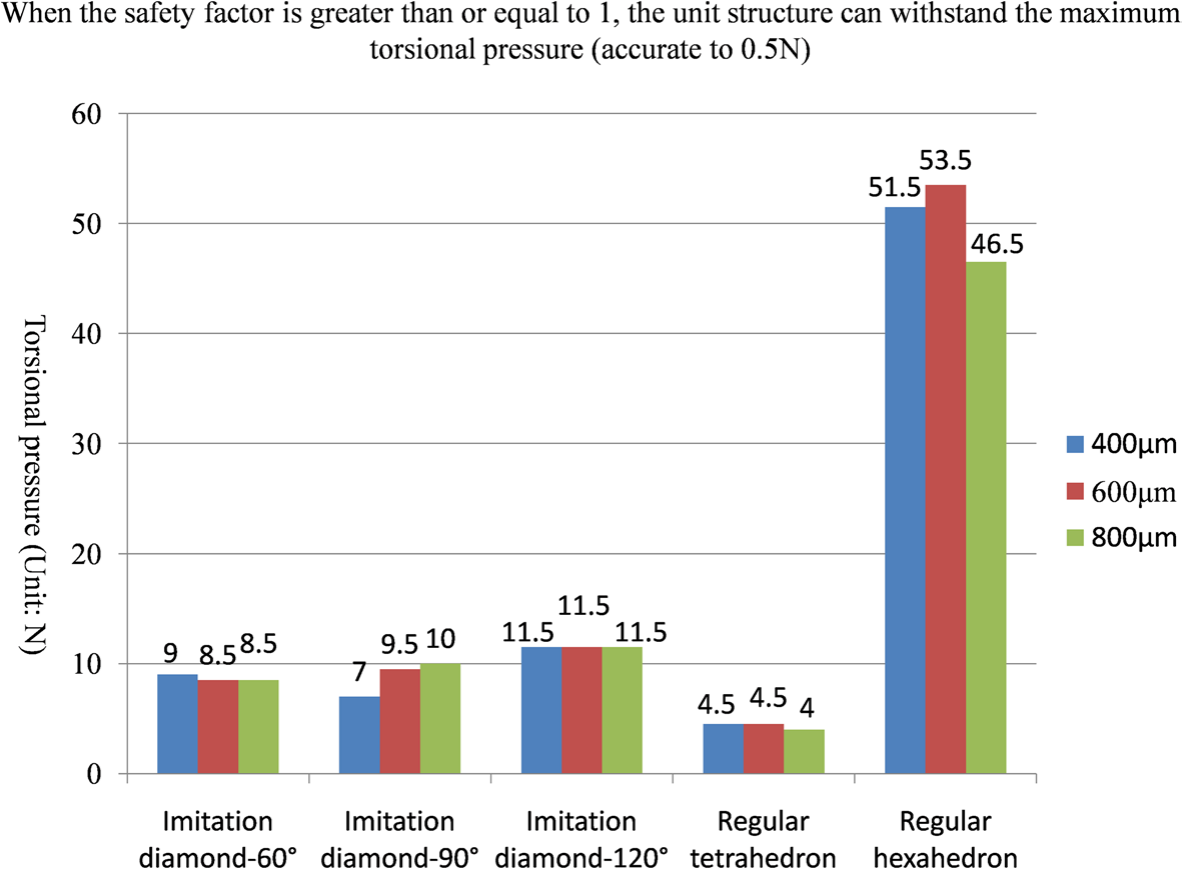
When the safety factor is greater than or equal to 1, the unit structure can withstand the maximum torsional pressure (accurate to 0.5 N)

The maximum positive pressure values that could be sustained on the fifteen kinds of unit structures in the safety state are shown in Fig. 5 and Table 1. The maximum lateral pressure values are shown in Fig. 6 and Table 2, and the maximum torsional pressure values are shown in Fig. 7 and Table 3. Through the analysis of the data in the table, the following conclusions can be drawn:

1. Under positive pressure, the maximum force of the imitation diamond and the regular hexahedron structure decreased with increasing pore size, the maximum force of the regular tetrahedron structure didn’t decrease with increasing pore size, and the maximum forces of the regular tetrahedron and the regular hexahedron structures were much larger than that of the imitation diamond structure, and the compressive strength of the regular hexahedron structure was the highest.
2. Under the lateral pressure, the maximum force of the five kinds of unit structures decreased with increasing pore size, and the maximum forces of the regular tetrahedron and regular hexahedron structures were much larger than that of the imitation diamond structure. The compressive strength of the regular hexahedron structure was the highest.
3. Under the torsional pressure, the maximum force of the imitation diamond-90° structure increased with increasing pore size. The maximum force of the other structure did not change with the pore size. The regular hexahedron structure had the greatest torsional resistance. And the regular tetrahedron structure had the smallest torsional resistance.
4. In the comprehensive analysis of positive pressures, the order of compressive capacity of the five types of unit models, from strong to weak, was regular hexahedron > regular tetrahedron > imitation diamond-120° > imitation diamond-90° > imitation diamond-60°.
5. In the lateral pressure comprehensive analysis, the order of the compressive capacity of the five types of unit models from strong to weak, was regular hexahedron > regular tetrahedron > imitation diamond-120° > imitation diamond-90° > imitation diamond-60°.
6. In the comprehensive analysis of the torsional pressure, the order of the compressive capacity of the five types of unit models, from strong to weak, was regular hexahedron > imitation diamond-120° > imitation diamond-90° > imitation diamond-60° > regular tetrahedron.

**Table 1:** When the safety factor is greater than or equal to 1, the unit structure can withstand the maximum positive pressure (accurate to 0.5 N)

Positive pressure (Unit: N)	400 μm	600 μm	800 μm
Imitation diamond-60°	10.5	8.0	6.0
Imitation diamond-90°	8.0	8.5	7.0
Imitation diamond-120°	11.0	9.0	7.5
Regular tetrahedron	88.5	92.0	93.0
Regular hexahedron	185.0	165.0	155.0

**Table 2:** When the safety factor is greater than or equal to 1, the unit structure can withstand the maximum lateral pressure (accurate to 0.5 N)

Lateral pressure (Unit: N)	400 μm	600 μm	800 μm
Imitation diamond-60°	3.0	2.0	1.5
Imitation diamond-90°	5.0	4.0	4.0
Imitation diamond-120°	8.5	7.5	6.0
Regular tetrahedron	65.0	45.0	39.5
Regular hexahedron	185.0	165.0	155.0

### Results and analyses of cylindrical model

The safety factors for the fifteen cylindrical models given the positive, lateral and torsional pressures are shown in Tables 4, 5, 6, 7 and 8. Through the analysis of the data in the tables, the following conclusions could be drawn:

1. Under the positive, lateral and torsional pressures, the safety factors of the imitation diamond, the regular tetrahedron and the regular hexahedron models decreased with increasing pore size.
2. Under positive pressure, considering factors such as the force, pore size and safety factor, the order of the compressive capacity of the five types of cylindrical models, from strong to weak, was regular hexahedron > regular tetrahedron > imitation diamond-90° > imitation diamond-60° > imitation diamond-120°.
3. Under the lateral pressure, considering factors such as the force, pore size and safety factor, the order of the compressive capacity of the five types of cylindrical models, from strong to weak, was regular hexahedron > regular tetrahedron > imitation diamond-120° > imitation diamond-90° > imitation diamond-60°.
4. Under the torsional pressure, considering factors such as the force, pore size and safety factor, the order of the compressive capacity of the five types of cylindrical models, from strong to weak, was regular hexahedron > imitation diamond-120° > imitation diamond-90° > regular tetrahedron > imitation diamond-60°.
5. Based on the above points, the order of the comprehensive compressive capacity of the five types of cylindrical models, from strong to weak, was regular hexahedron > regular tetrahedron > imitation diamond-120° > imitation diamond-90° > imitation diamond-60°. The smaller the pore size in each type of cylindrical model, the greater the compressive strength.

**Table 3:** When the safety factor is greater than or equal to 1, the unit structure can withstand the maximum torsional pressure (accurate to 0.5 N)

Torsional pressure (Unit: N)	400 μm	600 μm	800 μm
Imitation diamond-60°	9.0	8.5	8.5
Imitation diamond-90°	7.0	9.5	10.0
Imitation diamond-120°	11.5	11.5	11.5
Regular tetrahedron	4.5	4.5	4.0
Regular hexahedron	51.5	53.5	46.5

**Table 4:** Minimum safety factor for the imitation diamond-60° cylindrical model under different pressures

Minimum safety factor	Pore size (μm)	400	600	800
Positive pressure	600 N	2.63	0.96	0.81
	1800 N	0.88	0.33	0.05
	3000 N	0.53	0.18	0.16
Lateral pressure	600 N	0.30	0.25	0.15
	1800 N	0.10	0.08	0.05
Torsional pressure	2000 N	1.43	0.28	0.35
	3000 N	0.95	0.18	0.24
	4000 N	0.72	0.72	0.18

**Table 5:** Minimum safety factor for the imitation diamond-90° cylindrical model under different pressures

Minimum safety factor	Pore size (μm)	400	600	800
Positive pressure	600 N	2.74	1.19	1.58
	1800 N	0.91	0.40	0.53
	3000 N	0.55	0.24	0.32
Lateral pressure	600 N	0.59	0.81	0.40
	1800 N	0.20	0.27	0.13
Torsional pressure	2000 N	2.86	0.87	1.19
	3000 N	1.91	0.58	0.80
	4000 N	1.43	0.43	0.60

**Table 6:** Minimum safety factor for the imitation diamond-120° cylindrical model under different pressures

Minimum safety factor	Pore size (μm)	400	600	800
Positive pressure	600 N	1.47	1.64	0.69
	1800 N	0.49	0.55	0.23
	3000 N	0.29	0.33	0.14
Lateral pressure	600 N	1.43	1.40	1.14
	1800 N	0.48	0.47	0.38
Torsional pressure	2000 N	2.00	3.22	1.33
	3000 N	1.33	2.14	0.89
	4000 N	1.00	1.61	0.67

**Table 7:** Minimum safety factor for the regular tetrahedron cylindrical model under different pressures

Minimum safety factor	Pore size (μm)	400	600	800
Positive pressure	600 N	15.00	1.52	0.56
	1800 N	4.34	0.51	0.19
	3000 N	2.60	0.30	0.11
Lateral pressure	600 N	6.17	3.50	1.57
	1800 N	2.06	1.17	0.52
Torsional pressure	2000 N	1.99	0.94	0.49
	3000 N	1.33	0.63	0.32
	4000 N	1.00	0.47	0.24

**Table 8:** Minimum safety factor for the regular hexahedron cylindrical model under different pressures

Minimum safety factor	Pore size (μm)	400	600	800
Positive pressure	600 N	15.00	8.30	15.00
	1800 N	15.00	2.77	10.53
	3000 N	11.50	1.66	6.32
Lateral pressure	600 N	7.06	0.26	6.55
	1800 N	2.35	0.09	2.18
Torsional pressure	2000 N	5.69	0.79	8.27
	3000 N	3.80	0.36	8.27
	4000 N	3.80	0.27	4.14

## Discussion

Bone defects are common and the important problems to be solved in the clinic. Treatments of bone defects include autologous bone grafts, allogeneic bone grafts, bone cement and the implantation of artificial materials. At present, a variety of artificial materials have been used to treat bone defects, including metals because they have favorable characteristics. Ti6Al4V has good heat resistance, strength, plasticity, toughness, formability, corrosion resistance and biocompatibility. Compared to the space-holder technology [36], oaming method [37], and other methods, 3D printing technology can better control of the porosity, larger pore size [38], pore volume, spatial arrangement and other surface properties of scaffolds. The porous titanium scaffold made by 3D printing technology has good biomechanical properties, biocompatibility and lower elastic modulus [39] compared to titanium and its alloys.

The design of the pore morphology in 3D printed porous titanium scaffolds has a great influence on mechanics, and also affects the cell biologically. The factors that affect the growth of osteoblasts are not only pore size, and other factors such as connectivity, pore shape and porosity [40]. Therefore, the study of pore size and shape is important to the design of 3D printed porous titanium scaffolds. In this study, Autodesk Inventor software was used to design the unit structures and cylindrical models with different structures and different pore sizes. The lower limit of the pore size was 400 μm, which ensured that the pore size of the scaffold was large enough. There were five different types of unit structures, each with different shapes of pores. Any of the structures of the scaffold holes could be connected to any other. By finite element analyses, we found under positive pressure, the regular hexahedron cylindrical model was the strongest, then the regular tetrahedron cylindrical model, the imitation diamond cylindrical model was the worst, consistent with other studies [23, 30, 41]. Compared to other studies, we also carried out analysis under the lateral and torsional pressure, and found results were variably under different pressures, but the comprehensive compressive capacity of regular hexahedron cylindrical model was always the best under each pressure. This study showed the compressive strength of porous structures decreased with increases of pore size, similar to other studies [21, 42]. Most studies just analyzed cylindrical or cube models, the force analysis of the unit structure was also carried out in this study, and the results were consistent with those of the cylindrical models.

**Fig. 8:**
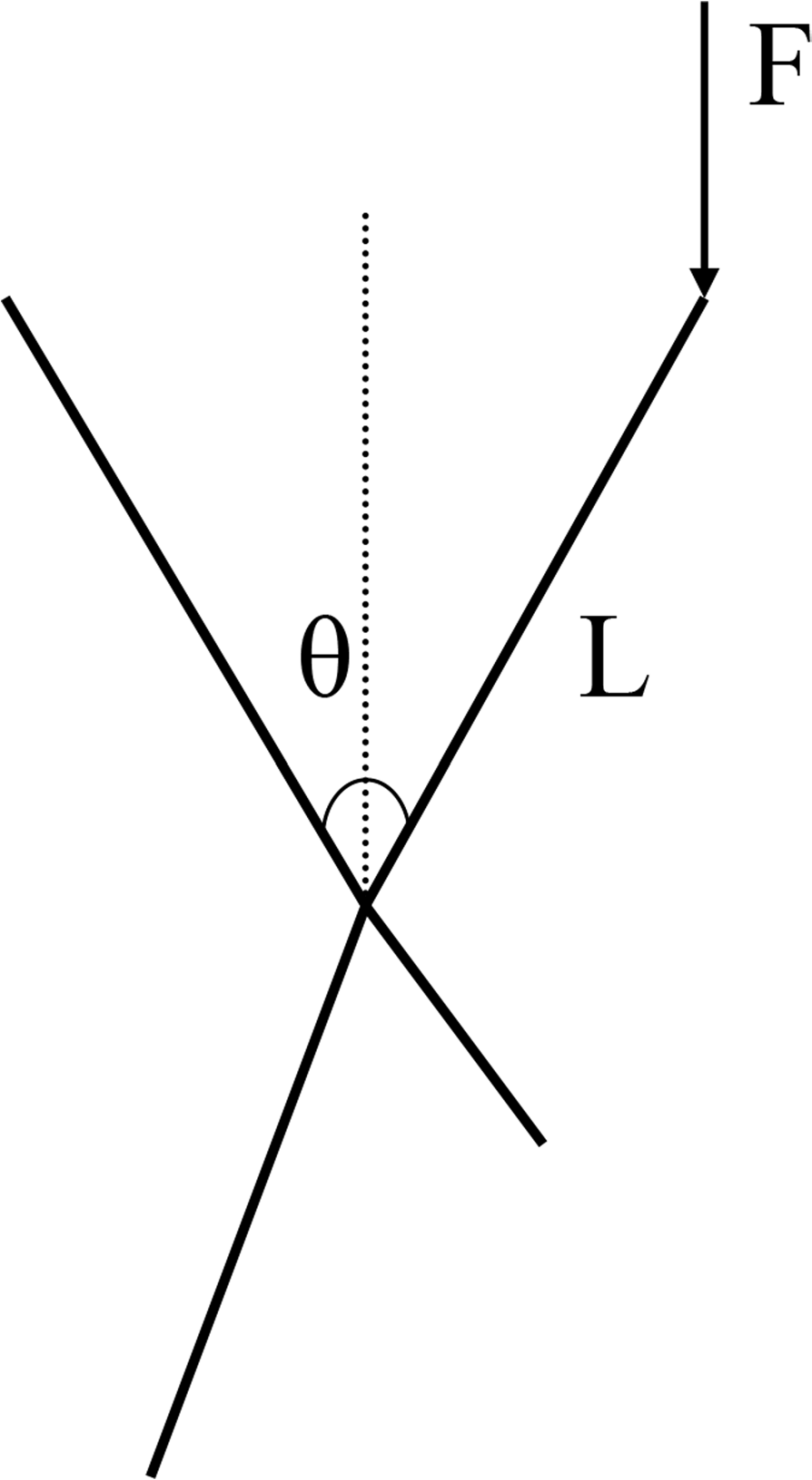
Positive pressure on the imitation diamond unit structure

The positive pressure for the imitation diamond unit structure could be analyzed with the simple model in Fig. 8.

The length of the bracket bar was L, the angle between the two bars was θ, the pressure on the bar end was F, the moment of the bracket center point was M, and the following formula could be obtained [23]:

$$\mathrm{M}=\mathrm{FLsin}\frac{\uptheta }{2}$$

From the above formula, when the moment M of the bracket center point was constant, the compressive strength at the point where the bracket was most vulnerable was constant; when θ was constant, L was larger and F was smaller. When L was constant, θ was larger (0° < θ < 180°) and F was smaller.

Therefore, for the imitation diamond structure, when the pore size was consistent, the angle θ was larger and the force F was smaller. When the angle between the two bars was constant, the pore size was larger, L was larger, and the force F was smaller. However, because the pore size of the imitation diamond was the same, θ was larger but L was smaller; therefore, it was not sufficient to use this formula for analysis.

For the regular tetrahedron, the vertex moment M was constant, while at the same time, there were three bars bearing pressure; thus compared to the imitation diamond structure, the regular tetrahedron could withstand greater force.

For the regular hexahedron, the force was in the longitudinal direction of the bar, which was the compressive strength of the titanium bar, and it could therefore withstand the greatest force.

The material of metallic biomaterials can affect the mechanical properties. The digital design and finite element analysis method are also applicable to other metallic biomaterials. Of course, the specific design is contingent on the properties of metallic biomaterials selected, especially the material can be made into finished products by 3D printing. The digital model can be obtained by input the relative physical properties data of the metallic biomaterial, design shapes and pore sizes when modeling, and finite element analysis can be carried out.

There are some shortcomings in this study: (1) Compared with dense titanium,3D printed porous titanium scaffolds have a lower modulus of elasticity, but this study did not analyze the elastic modulus. (2) This study did not measure or analyze the porosity, surface area or other factors. (3) The porous titanium scaffold used to fill knee bone defects is not simply under positive, lateral or torsional pressure, but may also be subject to various directions of the various pressures from various directions; this study failed to analyze this complex condition. (4) Due to the different pore sizes of unit structures contained in the fixed-size cylindrical model, the complete unit structure may not be retained at the edge of the model when intercepting, resulting in some data inconsistent with the theory. For example, in Table 8, when the 600 μm pore size models were subjected to force, the data obtained were not in the middle. (5) This study only used software to design and simulate forces; the obtained data are only a reference for further entity production and testing. Biocompatibility and osteoblast attachment, differentiation and growth on the 3D printed porous titanium scaffold require further studies.

## Conclusions

This study evaluated 3D printed porous titanium scaffolds with fifteen different pore structures under positive, lateral and torsional pressures. The order of the comprehensive compressive capacity of the five types of cylindrical models was regular hexahedron > regular tetrahedron > imitation diamond-120° > imitation diamond-90° > imitation diamond-60°, and for each type of cylinder, the smaller the pore size, the greater the compressive strength. The regular hexahedron, regular tetrahedron and imitation diamond-120° models appear to meet the conditions of large pore size and high compressive strength. In addition, the strength of each structure reduced when the pore size (400, 600 and 800 μm) increased. Obtaining the optimal design of the 3D printed porous titanium scaffold can provide a potentially effective clinical solution and ultimately promote the clinical application of titanium scaffolds.

## Data Availability

Materials described in the manuscript, including all relevant raw data, can be freely available to any scientist and reader wishing to use them for non-commercial purposes, without breaching participant confidentiality. The datasets used and/or analysed during the current study are available from the corresponding author on reasonable request. All data generated or analysed during this study are included in this published article.
